# Biomarkers Signal Contaminant Effects on the Organs of English Sole (*Parophrys vetulus*) from Puget Sound

**DOI:** 10.1289/ehp.8544

**Published:** 2006-02-02

**Authors:** Donald C. Malins, Katie M. Anderson, John J. Stegeman, Pawel Jaruga, Virginia M. Green, Naomi K. Gilman, Miral Dizdaroglu

**Affiliations:** 1 Biochemical Oncology Program, Pacific Northwest Research Institute, Seattle, Washington, USA; 2 Biology Department, Woods Hole Oceanographic Institution, Woods Hole, Massachusetts, USA; 3 Department of Chemical and Biochemical Engineering, University of Maryland Baltimore County, Baltimore, Maryland, USA; 4 Chemical Science and Technology Laboratory, National Institute of Standards and Technology, Gaithersburg, Maryland, USA

**Keywords:** cyclopurine nucleosides, cytochrome P4501A, DNA markers, DNA structure, Fourier transform–infrared spectroscopy, liquid chromatography/mass spectrometry

## Abstract

Fish living in contaminated environments accumulate toxic chemicals in their tissues. Biomarkers are needed to identify the resulting health effects, particularly focusing on early changes at a subcellular level. We used a suite of complementary biomarkers to signal contaminant-induced changes in the DNA structure and cellular physiology of the livers and gills of English sole (*Parophrys vetulus*). These sediment-dwelling fish were obtained from the industrialized lower Duwamish River (DR) in Seattle, Washington, and from Quartermaster Harbor (QMH), a relatively clean reference site in south Puget Sound. Fourier transform–infrared (FT-IR) spectroscopy, liquid chromatography/mass spectrometry (LC/MS), and gas chromatography/mass spectrometry (GC/MS) identified potentially deleterious alterations in the DNA structure of the DR fish livers and gills, compared with the QMH fish. Expression of CYP1A (a member of the cytochrome P450 multigene family of enzymes) signaled changes in the liver associated with the oxidation of organic xenobiotics, as previously found with the gill. The FT-IR models demonstrated that the liver DNA of the DR fish had a unique structure likely arising from exposure to environmental chemicals. Analysis by LC/MS and GC/MS showed higher concentrations of DNA base lesions in the liver DNA of the DR fish, suggesting that these base modifications contributed to this discrete DNA structure. A comparable analysis by LC/MS and GC/MS of base modifications provided similar results with the gill. The biomarkers described are highly promising for identifying contaminant-induced stresses in fish populations from polluted and reference sites and, in addition, for monitoring the progress of remedial actions.

The contamination of coastal environments poses an obvious threat to the health of a broad spectrum of phylogenetically diverse organisms. Consequently, a keen interest exists in finding ways to assess the health impacts on exposed populations of aquatic species (Barron et al. 2002; Myers et al. 1991). Biologic indicators play a prominent role in the assessment of toxic chemical exposures and effects (Ostrander 2005). One goal is the development of biomarkers for determining whether xenobiotics have altered the health of aquatic species at a contaminated site and whether remedial action (e.g., removal of contaminated sediments) has proven successful in reducing observed toxic effects (Cajaraville et al. 2000).

In an early discovery, Dawe et al. (1964) found neoplasia in the livers of wild fish from polluted sites. This discovery focused considerable attention on the health status of fish living in these environments. Subsequently, environmental chemical exposures were linked to other cellular lesions in fish livers (e.g., megalocytic hepatosis and foci of cellular alteration) (Malins et al. 1987; Moore and Myers 1994; Myers et al. 1991).

Refractory chemicals [e.g., polychlorinated biphenyls (PCBs)] that accumulate in the livers of fish produce hepatocellular carcinomas in rodents (Mayes et al. 1998). Polynuclear aromatic hydrocarbons (PAHs) (e.g., benzo[*a*]pyrene), which are readily metabolized in the livers of fish to carcinogenic metabolites, have been putatively linked to the development of liver tumors (Maccubbin 1994). Moreover, a number of subcellular alterations, such as in gene expression (Peterson and Bain 2004; Roling et al. 2004), and changes in immune response (Grinwis et al. 2000; Mondon et al. 2000) result from the intake of PAHs, PCBs, and other toxic organic compounds.

Field studies on the effects of sediment contamination on bottom-dwelling English sole (*Parophrys vetulus*) at multiple Puget Sound sites (Washington State) were conducted between 1979 and 1985. These studies revealed significantly higher concentrations of toxic chemicals in Seattle’s industrialized Duwamish River (DR), notably PAHs and PCBs, compared with “clean” reference sites (e.g., Case Inlet) (Malins et al. 1987). Hepatic neoplasms (~ 17% prevalence) were revealed in fish from urban (industrialized) sites, such as the DR, but not in fish from nonurban (essentially nonindustrialized) reference sites (Malins et al. 1987). Noncancerous idiopathic lesions (e.g., foci of cellular alteration) were found primarily in fish from the DR, compared with the reference sites (Malins et al. 1987). In 2001, the state of the environment in the DR caused the U.S. Environmental Protection Agency (EPA) to add the Lower Duwamish Waterway to the National Priorities List (Superfund) because of sediment contamination (U.S. EPA 2001).

Factors giving rise to differences in biologic effects between the DR and reference sites are obviously complex and multifaceted. Reactive oxygen species [ROS; e.g., the hydroxyl radical (**·**OH)] likely contributed to these differences by reacting with and thus damaging the DNA bases of fish exposed to toxic chemicals (Malins et al. 1996). This damage has been associated with an increased risk for tumor formation. For example, a previous comparative study of liver DNA in English sole from the DR in 1993 and 1995 and in Quartermaster Harbor (QMH), a relatively clean location in south Puget Sound, in 1995 showed higher concentrations of the mutagenic base lesions 8-hydroxyguanine (8-OH-G) and 8-hydroxy-adenine (8-OH-A) in the DR fish (Malins et al. 1996).

Fourier transform–infrared (FT-IR) spectral analysis showed that the DNA structures of the livers from the DR 1993 and DR 1995 groups differed significantly from those in the QMH 1995 group (Malins et al. 1997). Furthermore, there were structural differences in the liver DNA from the DR fish between 1993 and 1995, likely reflecting alterations in the nature and degree of the environmental contamination that occurred during this 2-year period. Compared with the DR fish, the liver DNA from the QMH 1995 group exhibited a remarkable degree of structural homogeneity, implying that these fish had suffered virtually no evincible DNA damage (Malins et al. 1997). Notably, the high degree of heterogeneity (variance) found between the individual DNA structures of the two DR groups decreased appreciably between 1993 and 1995, suggesting a change likely symptomatic of a reduction in DNA damage, perhaps associated with sediment remediation. The spectral/structural differences in the liver DNA of the DR groups were shown to be related to alterations in the C–O stretching and NH_2_ bending vibrations of the nucleic acids, and to stretching vibrations of the phosphodiester–deoxyribose backbone (Malins et al. 1997).

Most recently, we compared the effects of waterborne toxic chemicals on the gill DNA of English sole obtained from the DR and QMH in October 2000 (Malins et al. 2004c). The biomarker systems used for evaluating changes at the subcellular level were FT-IR statistical models of DNA structure (Malins et al. 2004c, 2005) and cytochrome P4501A (CYP1A) expression (Miller et al. 2004; Stegeman et al. 2001). Xenobiotic-induced changes in DNA are known to be associated with many genetic and other diseases in aquatic organisms (Maccubbin 1994). Moreover, increased expression of CYP1A indicates exposure to aromatic hydrocarbon receptor agonists and increases the rate of oxidative modifications in complex mixtures of toxic chemicals (e.g., PAHs and planar PCB congeners) in fish tissues (White et al. 1997). Thus, these complementary biomarkers are valuable for signaling the risk for adverse health effects by comparing results from contaminated and reference fish populations.

Using the FT-IR models, we developed a DNA damage index that discriminated between the contaminated and reference fish groups based on structural differences in the gill DNA that likely resulted from exposure of the DR fish to waterborne contaminants (Malins et al. 2004c). These findings were corroborated by significantly higher CYP1A expression in the gills of the DR fish, as well as by the presence of histologically evinced lesions (e.g., foci of necrosis and epithelial hyperplasia of the gill lamellae) that were essentially absent in the QMH fish.

In this article, we describe the results of a study of the same group of English sole used in the previous gill work. In addition to the two biomarkers used for identifying subcellular changes in the gill, we have now found damage to the base structure of the liver and gill DNA using liquid chromatography/mass spectrometry (LC/MS) and gas chromatography/mass spectrometry (GC/MS). Although previously used with mammalian tissues (Dizdaroglu et al. 2001; Jaruga et al. 2002, 2004), to our knowledge this is the first reported use of LC/MS for evaluating differences between DNA base lesion concentrations in aquatic organisms from test and reference sites.

## Materials and Methods

### English sole tissues

The liver and gills of English sole from the lower DR (*n* = 18; 141 ± 70 g) and QMH (*n* = 18; 183 ± 79 g) in October 2000 (Malins et al. 2004c) were provided by Applied Marine Sciences (Livermore, CA). Sections of liver tissue were placed in formalin for histologic examination. The remaining liver samples were kept frozen (–80°C). The gill tissues were treated similarly, as previously reported (Malins et al. 2004c).

### CYP1A expression and histology

Levels of CYP1A expression were determined using immunohistochemical techniques (Van Veld et al. 1997; Woodin et al. 1997). Histologic changes in the liver were identified as previously described (Malins et al. 1987; Moore and Myers 1994; Myers et al. 1991).

### DNA extraction

As previously reported (Malins et al. 2004c, 2005), approximately 300 μg DNA was extracted from each tissue sample (~ 350 mg: DR, *n* = 10; QMH, *n* = 10) with 500/G Genomic-tips (Qiagen, Chatsworth, CA) using the recommended protocol. DNA, in a buffer solution, was passed through a 5.0 μm Cameo 30N filter (Osmonics, Minnetonka, MN) at room temperature before precipitation and then washed with ice-cold 70% ethanol. This mild extraction procedure, conducted on test and reference samples under identical conditions, essentially precludes the possibility that base oxidation would influence the reported comparative studies. Before FT-IR spectral analysis, the DNA was dissolved in 10–40 μL Optima-grade distilled water (Fisher Scientific, Hampton, NH).

### Analysis by FT-IR spectroscopy

We analyzed the liver DNA using previously reported protocols (Malins et al. 2004c, 2005). A 0.2 μL aliquot of DNA solution was spotted on a BaF_2_ plate. As the spot dried, an outer ring of DNA was formed. Two separate spots were formed for each DNA sample. The rings of DNA were lyophilized to complete dryness. Using an FT-IR microscope spectrometer (System 2000; PerkinElmer, Wellesley, MA), we made 20 spectral measurements randomly around each of the two rings per sample. Resulting spectral measurements were expressed as percent transmittance, which was converted (Fourier-transformed) into absorbance. Each spectrum was baselined and normalized between 1,300 and 760 cm^−1^. The remaining DNA was lyophilized for subsequent base lesion analysis.

### Analysis by LC/MS

We used LC/MS with isotope-dilution to identify and quantify 8-hydroxy-2′-deoxyguanosine (8-OH-dG), 8-hydroxy-2′-deoxyadenosine (8-OH-dA), (5′ S )-8, 5 ′- cyclo - 2 ′- deoxyguanosine [(5′S)-cdG], and (5′S)-8,5′cyclo-2′-deoxy-adenosine [(5′S)-cdA] in the liver and gill DNA samples. A stable isotope-labeled analog of 8-OH-dG (8-OH-dG-^15^N_5_) purchased from Cambridge Isotope Laboratories (Cambridge, MA) was used as an internal standard. Stable isotope-labeled analogues of 8-OH-dA, (5′S)-cdG, and (5′S)-cdA [8-OH-dA-^15^N_5_, (5′S)-cdG-^15^N_5_, and (5′S)-cdA-15N_5_, respectively] were prepared as described (Jaruga et al. 2002, 2004) and used as internal standards. Aliquots (50 μg) of DNA were supplemented with aliquots of internal standards; hydrolyzed with nuclease P1, snake venom phosphodiesterase, and alkaline phosphatase for 24 hr; and then analyzed by LC/MS as described by Jaruga et al. (2004). For identification and quantification, selected ion monitoring served to monitor the characteristic ions of the modified nucleosides and their stable isotope-labeled analogues.

### Analysis by GC/MS

2,6-Diamino-4-hydroxy-5-formamidopyrimidine (FapyG) and 4,6-diamino-5-formamidopyrimidine (FapyA) were identified and quantified using GC/MS with isotope-dilution after hydrolysis of DNA samples with *Escherichia coli* Fpg protein to release FapyG and FapyA. Fpg was isolated as previously described (Reddy et al. 2004). Stable isotope-labeled analogues of FapyG and FapyA (FapyG-^13^C,^15^N_2_ and FapyA-^13^C,^15^N_2_) were purchased from Cambridge Isotope Laboratories. Aliquots (50 μg) of DNA were supplemented with aliquots of the internal standards FapyG-^13^C,^15^N_2_ and FapyA-^13^C,^15^N_2_ and hydrolyzed with 2 μg Fpg. The hydrolysates were trimethylsilylated and then analyzed by GC/MS as described by Reddy et al. (2004). For identification and quantification, we used selected ion monitoring to monitor the characteristic ions of the trimethylsilylated FapyG and FapyA and their stable isotope-labeled analogues.

### Statistical analyses

For FT-IR spectral data, we performed a *t*-test to establish statistical differences (*p*-values) between the mean DNA spectra for each fish group at each wavenumber. Although *p*-values over the range of wavenumbers were not statistically independent, spectral regions with *p* < 0.05 likely represent actual structural differences between groups (Malins et al. 2000).

We conducted principal components analysis (PCA) as reported previously (Malins et al. 2005). PCA involves approximately 10^6^ correlations between spectral absorbances and integrates differences in peak heights, peak locations, and various combinations thereof. This statistical procedure was undertaken on the spectrum of each sample, resulting in 10 principal component (PC) scores per sample. Using *t*-tests, significant differences (*p* < 0.05) in PC scores were determined between groups. PCs showing the most significant differences were used to construct a three-dimensional plot. Logistic regression analysis, using a highly significant PC, was the basis for establishing a DNA damage index (Malins et al. 2004c, 2005) that reflected differences in the unique spectral properties of the liver DNA for each fish.

For LC/MS and GC/MS data, the following statistical procedures provided comparative information on differences in base lesion concentrations in the liver and gills of fish from the contaminated (DR) and reference (QMH) sites. We used *t*-tests to determine statistical differences (*p*-values) between groups for each base lesion. A Levene’s test was used to identify significant differences (*p*-values) in the variance (a measure of variability around the mean) of each group for base lesion concentrations (Malins et al. 2002).

## Results and Discussion

### Histology

We identified several idiopathic hepatic lesions in the DR fish. The most prominent lesions were basophilia and macrophage aggregates, which were found in 10 and 6 of the samples, respectively. However, these lesions were detected in only 1 and 2 of the QMH samples, respectively (Table 1). These findings are comparable with those previously obtained in the gills of these fish groups (Malins et al. 2004c) in which all the DR fish and half the QMH fish exhibited idiopathic lesions. These changes in the liver would be expected to arise preferentially from the intake of toxic substances in food (e.g., sediment invertebrates), whereas the changes in the gills likely resulted from exposure to water-borne chemicals that are readily transferred across the gills (e.g., low-molecular-weight organic compounds, such as alkylated phenols) (Randall et al. 1996). However, these routes of exposure are not mutually exclusive in that many toxic chemicals absorbed through the gills are ultimately destined for the liver. Comparative data on the gill histology have been reported previously (Malins et al. 2004c).

### CYP1A expression

**Figure 1:**
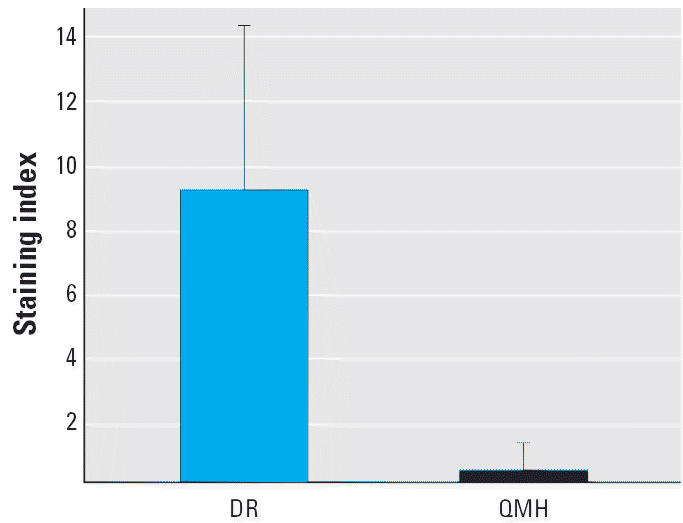
Mean ± SD for CYP1A staining of the liver from each fish group. Seventeen of 19 DR samples and 3 of 16 QMH samples stained positive.

CYP1A staining in the livers of the DR fish (9.3 ± 5.2) was approximately 25-fold greater than in the QMH fish (0.4 ± 0.9) (Figure 1). This high degree of CYP1A expression is very likely associated with the accumulation of comparatively high concentrations of toxic chemicals, such as PAHs and planar PCB congeners, aryl hydrocarbon receptor agonists that occur in the DR and that are known to induce CYP1A expression (Stegeman et al. 2001). These findings are consistent with evidence demonstrating that CYP1A expression in the liver is a useful indicator of contaminant exposure in fish (Smolowitz et al. 1992; White et al. 1997). The previously reported high degree of CYP1A staining in the gill epithelium of these DR fish, compared with the low level found in the QMH fish, is consistent with the present findings.

### Analysis by FT-IR spectroscopy

FT-IR spectroscopy can provide information on alterations in the nucleotide base structure and vertical base stacking interactions as well as on conformational changes in the phosphodiester–deoxyribose backbone (Malins et al. 2000; Parker 1983; Tsuboi 1969). A unique advantage of this technology is that it identifies subtle changes in DNA structure from various endogenous and exogenous chemical stresses. FT-IR spectral analysis is an attractive technique for revealing early changes in DNA believed to signal a high risk for tumor formation in humans (Malins et al. 2004b) and rodents (Malins et al. 2004a), as well as in aquatic organisms (Malins et al. 1997, 2004c).

**Figure 2:**
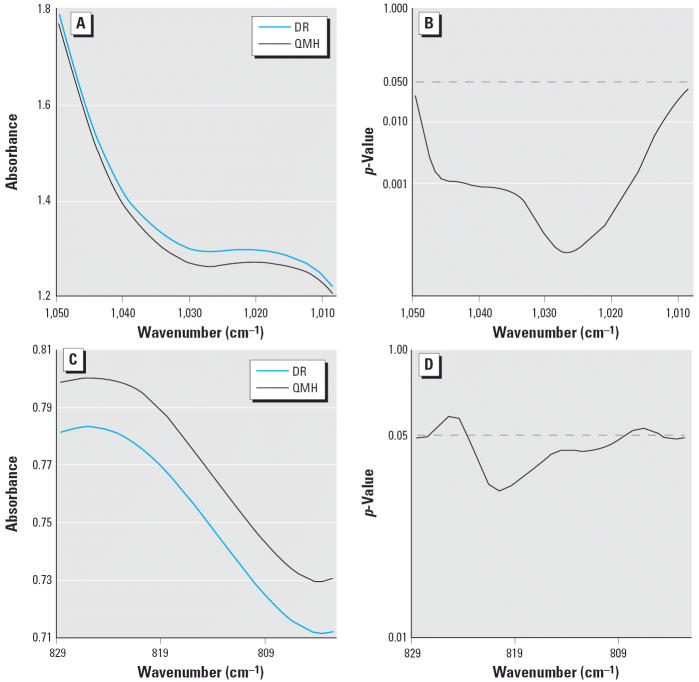
(*A*) Comparison of mean DNA spectra of liver from the DR and QMH fish between 1,050 and 1,009 cm^−1^. (*B*) *p*-Values from a *t*-test showing significant differences between these mean spectra. (*C*) Comparison of mean DNA spectra of liver from the DR and QMH fish between 829 and 803 cm^−1^. (*D*) *p*-Values from a *t*-test showing significant differences between these mean spectra.

In comparing the mean DNA spectra of the DR and QMH fish livers, we found significant differences between the groups spanning 10.4% of the spectral range (1,300–700 cm^−1^), indicating differences in backbone structure. This percentage is about twice that expected by chance (Malins et al. 2000). No spectral evidence was found for differences in the nucleotide base structure, which is assigned to approximately 1,750–1,300 cm^−1^. The comparison of spectral means from 1,050–1,009 cm^−1^ and 829–803 cm^−1^ with the corresponding *p*-values is shown in Figure 2. Spectral differences, such as at approximately 1,025 cm^−1^ (Figure 2A,B) and between approximately 829 and 803 cm^−1^ (Figure 2C,D), are attributed to ribose–phosphate main-chain vibrations (Tsuboi 1969).

**Figure 3:**
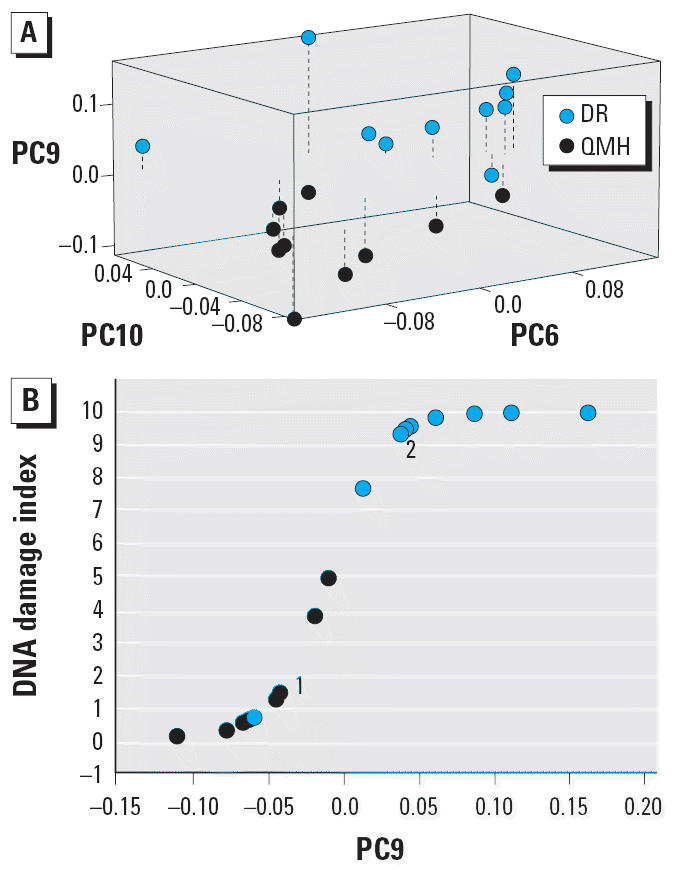
(*A*) Three-dimensional separation of PC scores from the DNA spectra of liver from the DR and QMH fish. Dashed lines show the distance from the PC9 baseline level of 0. (*B*) DNA damage index derived by logistic regression analysis using PC9 scores for liver DNA from the DR and QMH fish. Overlapping points: 1, Two QMH samples; 2, two DR samples.

PCA of the DNA spectra from the DR and QMH liver samples yielded four PCs (*p* ≤ 0.01), of which PC6, PC9, and PC10 were the most statistically significant (*p* ≤ 0.001). These PCs were used to construct a three-dimensional plot (Figure 3A). The plot demonstrates that the samples from each group clustered in different regions of three-dimensional space. This virtually complete separation establishes that each group had a unique DNA structure.

The separation of the DR and QMH liver DNA into two structurally distinct groups (Figure 3A) led us to conduct a logistic regression analysis on the spectral data. This analysis provided the basis for establishing a DNA damage index (Malins et al. 2004c) using PC9, the most significant PC (*p* < 0.001). Strikingly, the resulting damage index (Figure 3B) had a 90% probability for correctly identifying a DR sample and a 100% probability for correctly identifying a QMH sample.

In the present study of the liver DNA, structural differences between the DR and QMH groups were only found in the spectral region associated with the backbone (1,300–700 cm^−1^). In contrast, in the gill study, the DNA structural differences were restricted to the spectral area associated with the nucleotide bases (1,750–1,300 cm^−1^) (Malins et al. 2004c). The results of the two studies were quite similar with respect to the high probability of correctly differentiating between the fish exposed to toxic chemicals and those from a relatively clean environment. The highly discriminating power of the DNA damage index, first used with the gill DNA (Malins et al. 2004c) and now with the liver, suggests that such an index holds considerable promise for identifying DNA damage in different tissues from aquatic organisms exposed to environmental chemicals.

### Analysis by LC/MS and GC/MS

Living cells are continually exposed to damaging ROS arising from normal cellular metabolism or from one-electron oxidations of xenobiotics. Notably, planar PCB congeners stimulate release of ROS from induced fish liver microsomes, ostensibly by uncoupling the CYP1A catalytic cycles (Schlezinger et al. 1999). The liver is a prominent site for these reactions, as demonstrated with aquatic vertebrates (Maccubbin 1994; Malins et al. 1996). The highly reactive **·**OH, generated from the superoxide radical and H_2_O_2_ via metal ion catalysis (Imlay et al. 1988), reacts with guanine and adenine to produce redox-ambivalent 8-OH–adduct radicals (Candeias and Steenken 2000; Steenken 1989). These intermediate radicals are converted oxidatively to mutagenic 8-OH purines (i.e., 8-OH-G and 8-OH-A) and reductively to mutagenic formamidopyrimidines (i.e., FapyG and FapyA) (Gajewski et al. 1990; Steenken 1989). More than 30 of these types of base lesions have been identified in tissues from different terrestrial species (Dizdaroglu 1992) and in the livers of fish from contaminated environments (Malins et al. 1996).

The 8,5′-cyclopurine-2′-deoxynucleosides are an additional class of oxidatively induced DNA lesions that have been found previously in human tissues (Dizdaroglu et al. 2001; Jaruga et al. 2002, 2004). These cyclopurine nucleosides result from abstraction of an H from C-5′ of 2′-deoxyribose by the **·**OH, followed by cyclization of the resulting sugar radical onto the C-8 position of the purine of the same nucleoside, and ultimately oxidation of the resulting radical (Dizdaroglu 1986; Dizdaroglu et al. 2002). Cyclopurine nucleosides are considered tandem lesions in that concomitant damage is produced in both the base moieties and the sugar (Dizdaroglu 1986).

**Figure 4:**
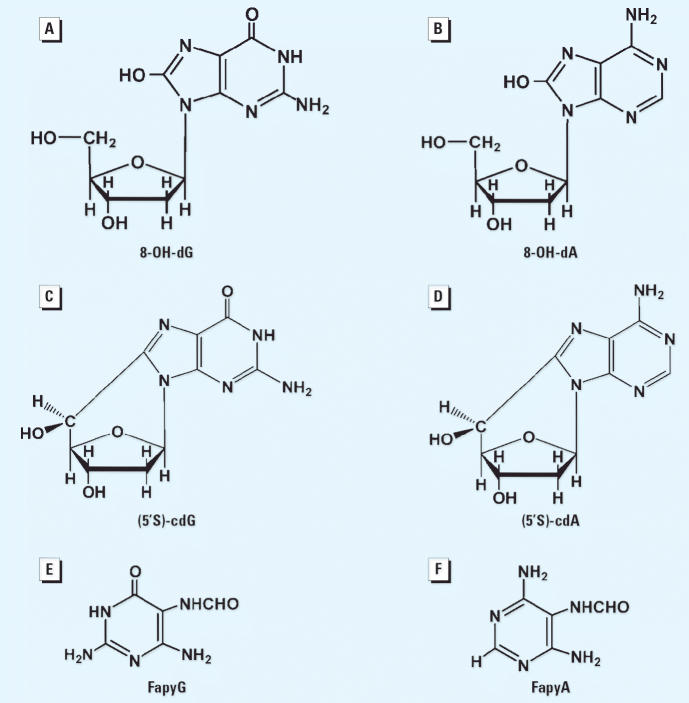
Chemical structures identified in the liver and gill DNA of the DR and QMH fish. (*A*) 8-OH-dG. (*B*) 8-OH-dA. (*C*) (5’S)-cdG. (*D*) (5’S)-cdA. (*E*) FapyG. (*F*) FapyA.

**Figure 5:**
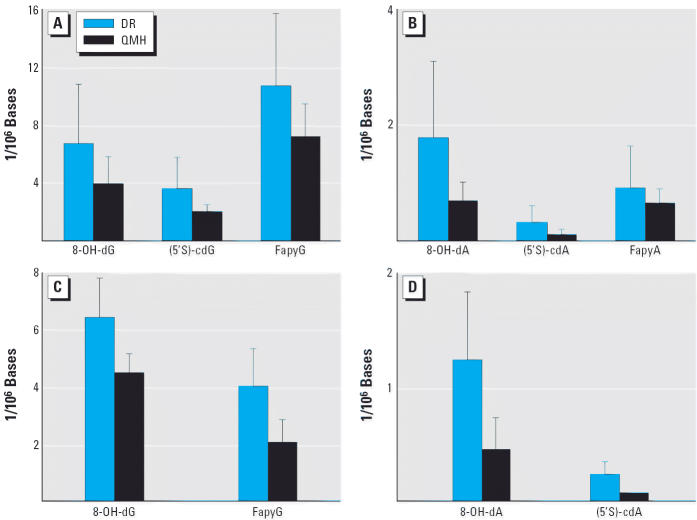
Concentrations of base modifications in liver DNA (*A* and *B*) and gill DNA (*C* and *D*) of the DR and QMH fish.

Analyses by LC/MS of the liver DNA from the DR and QMH fish revealed the presence of 8-OH-dG and 8-OH-dA, as well as the analogous cyclopurine nucleosides (Figure 4). This comparative study revealed that the DR fish had significantly (*p* ≤ 0.05) higher DNA concentrations of (5′S)-cdG (Figure 5A), 8-OH-dA, and (5′S)-cdA (Figure 5B). GC/MS was used to measure FapyG and FapyA (Figure 4). Concentrations of 8-OH-dG, FapyG (Figure 5A), and FapyA (Figure 5B) were also higher in the DR fish than in the QMH group, but the differences were not statistically significant (*p* > 0.05). Although the DNA base lesion concentrations of the liver were higher in the DR compared with the QMH fish, the actual levels (parts per million) were too low to be detected by FT-IR spectroscopy. Four of the base lesion concentrations in the gill DNA were also significantly (*p* < 0.01) higher in the DR fish compared with the QMH fish (Figure 5C,D). The concentrations of (5′S)-cdG and FapyA were too low to measure.

In a previous study using GC/MS, we compared concentrations of 8-OH-G and 8-OH-A in the liver DNA of English sole obtained from the DR in 1993 and 1995 and from QMH in 1995 (Malins et al. 1996). In 1993, the concentration of 8-OH-A was eight times higher in the DR fish compared with the reference fish (*p* < 0.001), and the concentration of 8-OH-G was four times higher (*p* = 0.01). Further, FT-IR spectral differences were demonstrated in both the DNA base and backbone structures.

The present base lesion comparisons by LC/MS and GC/MS clearly demonstrate that about 7 years later, the liver DNA of fish from the DR still show a considerably higher degree of oxidative damage compared with the reference fish. Although the lower base lesion levels presently obtained are not surprising because of pollution controls affecting the contaminant status of the DR (U.S. EPA 2001), any direct comparison of the 1993 and 1995 data with the results obtained in 2000 would be questionable in that the earlier findings involved phenol/chloroform extraction of DNA.

Statistically higher variances for the base lesion concentrations were found with the DR liver DNA compared with those of the QMH samples, ranging from approximately 5-fold for the 8-OH-dG (*p* = 0.03) to 30-fold for the (5′S)-cdG (*p* < 0.01) (Table 2). The consistently greater variance in the DR samples implies more heterogeneity in the nucleotide base structures compared with the QMH group. With the exception of FapyG, the variance of the base lesions concentrations was also significantly (*p* ≤ 0.03) higher in the gill DNA of the DR fish (Table 2). The higher degree of variance in the DR group is most likely attributable to reactions of xenobiotics and/or metabolites with DNA, thus disrupting the normal architecture of this biopolymer and creating a variety of oxidation products. These findings suggest that variance in the DNA base structure of fish tissues is a potentially useful new biomarker for signaling chemical-contaminant–induced alterations at the population level.

## Conclusions

FT-IR statistical models demonstrated the ability to differentiate between the DR and QMH fish based on the unique DNA structure of each group. These unique structural characteristics were the means for establishing a DNA damage index, which was shown to be an effective indicator of contaminant-induced damage to liver DNA. These results are comparable with those previously obtained with the gills of these fish (Malins et al. 2004c) and suggest that the damage index has the potential to be used with a variety of other tissues. The changes in the liver DNA likely reflect contamination primarily through the diet, whereas the changes in the gill DNA probably mostly reflect exposure to waterborne chemicals. The DNA damage index can thus provide insight into routes of contamination and their relative impacts on cellular physiology.

PC plots derived from FT-IR spectra of liver DNA of English sole obtained from the DR in 1993 and 1995 showed that the DNA structures were readily distinguishable from each other, as well as from the DNA of the QMH reference fish (Malins et al. 1997). In the present study, the liver DNA of the DR fish is still structurally different from that of the QMH fish (implying the continued presence of xenobiotic-induced DNA damage), despite many years of sediment cleanups and efforts to control the input of toxic chemicals (Lower Duwamish Waterway Group 2004; Mickelson and McElhany 2002).

The differences in base lesion concentrations found in the liver and gill DNA between the fish from the DR and QMH provide useful biomarker information on reactions resulting in base oxidations. The discovery that the cyclopurine nucleosides accumulated in relatively higher concentrations in the DNA of the DR fish adds a new dimension to previous studies using the 8-OH-G lesions as biomarkers (Malins et al. 1996). Cyclopurine nucleosides are likely to be removed in living cells by nucleotide excision repair (NER), rather than by base excision repair because of the presence of the covalent bond between the sugar and base moieties, as suggested previously (Dizdaroglu 1986) and evidenced by new data (Brooks 2002).

Cyclopurine nucleosides would be expected to induce alterations in the conformational structure of the DNA backbone as a consequence of the covalent bond formed between the C-8 of purines and C′-5 of the deoxyribose moiety. Such a distortion may partially account for the uniquely modified structure of the liver DNA from the DR fish (as evinced by FT-IR spectroscopy), which likely arose from exposure to toxic chemicals in the environment. Although little is known about the biologic significance of the cyclopurine nucleosides, they may alter the fidelity of transcription and replication, thus increasing the risk for disease states (e.g., as identified in the liver; Table 1), especially in NER-deficient cells.

Further information is needed on the health implications of cyclopurine nucleosides in fish; however, several studies suggest that they might be detrimental to the viability of living cells, such as reflected in NER-deficient xeroderma pigmentosum (Brooks 2002). The results reported here provide an additional perspective of the effects of ROS on nucleotide base structure by showing that the levels of cyclopurine nucleosides are higher in fish exposed to contaminants than those from clean areas. Thus, we suggest the use of cyclopurine nucleosides as novel biomarkers for identifying xenobiotic-induced changes in DNA.

The results obtained from the analyses of FT-IR, LC/MS, and GC/MS data were consistent with the findings for CYP1A expression and liver histology. Clearly, the choice of one or more biomarker systems for assessing contaminant effects at a particular site will likely depend on the nature and degree of the environmental contamination.

The discovery that the variance in the 8-OH-dG, 8-OH-dA, FapyG, FapyA, and cyclopurine nucleosides was substantially higher in the DNA of the DR fish suggests that variance is also an attractive biomarker for identifying pollution effects at the population level. Moreover, the information on DNA structure obtained by FT-IR spectroscopy, complemented by LC/MS and GC/MS, has the advantage of providing an assessment of contaminant effects on various tissues in aquatic species. The differences found in DNA structure between the DR and QMH fish illustrate the potential that the biomarker systems described have for monitoring and evaluating the efficacy of environmental remediation.

## Figures and Tables

**Table 1:** Number of English sole with liver lesions.

Condition	DR (*n* = 18)	QMH (*n* = 18)
Basophilia	10	1
Macrophage aggregates	6	2
Spongiosis hepatis	3	0
Foci of cellular alteration	2	0

**Table 2:** Variance differences in base lesion concentrations in the DNA from the DR and QMH fish livers and gills (expressed as base lesions/10^6^ nucleosides).

Tissue	8-OH-dG	8-OH-dA	(5′S)-cdG	(5′S)-cdA	FapyG	FapyA
Liver						
DR variance	16.88	1.73	4.43	0.08	25.12	0.54
QMH variance	3.39	0.10	0.15	0.01	5.10	0.05
Levene’s test	0.03	0.00	0.00	0.00	0.03	0.04
Gill						
DR variance	1.76	0.33	ND	0.01	1.63	ND
QMH variance	0.39	0.07	ND	0.00	0.62	ND
Levene’s test	0.03	0.04	ND	0.00	0.16	ND

ND, not determined.
